# 

**Published:** 1858-09

**Authors:** 


### CONTENTS.

ORIGINAL ESSAYS AND COMMUNICATIONS.

Filling Teeth, By J. Taft,........................................................ 5
How are Good Teeth Secured ? By E. Collins,........................................ 12
Irregularities in the Number of Teeth, By W. H. Shadoan,.............................. 16
Dental Quacks,........................................................................ 18
Remarks on the best means of securing good Teeth. Read before the "American Dental Convention," Cincinnati, By J. Richardson,........................................ 24
Blindness caused by Engorgement of the Maxillary Sinus. Read before the Medical Society of Beaver County, By J. Murray, Surgeon Dentist, Beaver, Pa.,.................. 32
Address on Artificial Dentures, By Dr. J. Allen,...................................... 35
Lateral Pressure of the Teeth considered in relation to obsecure Nervous affections. Read at the Fourth Annual Meeting of the "American Dental Convention, By J. M'Calla. 43

PROCEEDINGS OF SOCIETIES.

American Dental Convention—Fourth Annual Session,..................................... 61
Fourth Annual Meeting of the American Dental Convention. Discussions,................. 70

Miscellaneous......................................................................... 104
Editorial............................................................................. 119

